# The role of Na^+^‐K^+^‐ATPase in the epileptic brain

**DOI:** 10.1111/cns.13893

**Published:** 2022-06-25

**Authors:** Jinyi Sun, Yang Zheng, Zhong Chen, Yi Wang

**Affiliations:** ^1^ Institute of Pharmacology & Toxicology, College of Pharmaceutical Sciences Zhejiang University Hangzhou China; ^2^ Key Laboratory of Neuropharmacology and Translational Medicine of Zhejiang Province, School of Pharmaceutical Sciences Zhejiang Chinese Medical University Hangzhou China; ^3^ Epilepsy Center, Department of Neurology, Second Affiliated Hospital, School of Medicine Zhejiang University Hangzhou China

**Keywords:** drug target, epilepsy, excitability, Na+‐K+‐ATPase

## Abstract

Na+‐K+‐ATPase, a P‐type ATP‐powered ion transporter on cell membrane, plays a vital role in cellular excitability. Cellular hyperexcitability, accompanied by hypersynchronous firing, is an important basis for seizures/epilepsy. An increasing number of studies point to a significant contribution of Na+‐K+‐ATPase to epilepsy, although discordant results exist. In this review, we comprehensively summarize the structure and physiological function of Na+‐K+‐ATPase in the central nervous system and critically evaluate the role of Na+‐K+‐ATPase in the epileptic brain. Importantly, we further provide perspectives on some possible research directions and discuss its potential as a therapeutic target for the treatment of epilepsy.

## Graphical abstract

In this review, we comprehensively summarize the structure and physiological function of Na+‐K+‐ATPase in neuronal excitability of central nervous system and evaluate the role of Na+‐K+‐ATPase in epileptic brain. Importantly, we further provide perspectives on some possible research directions and discuss its potential as a therapeutic target for the treatment of epilepsy.

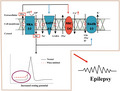

## 1 INTRODUCTION

Na+‐K+‐ATPase, discovered by Skou,^1^ is one of the most crucial Adenosine‐5′‐Triphosphate (ATP)‐powered P‐type ion transporters. It is essential for normal structure and function of all cell membranes. Its primary functions include maintenance ion gradient across membranes^2^, ^3^, ^4^ and uptake and release of neurotransmitters.^5^, ^6^, ^7^ Therefore, Na+‐K+‐ATPase plays a crucial role in ion homeostasis and cellular excitability. Dysfunction of Na+‐K+‐ATPase may lead to many types of central nervous system (CNS) disorders, including epilepsy.^8^, ^9^, ^10^, ^11^

Epilepsy is a common neurological disorder characterized by recurrent spontaneous seizures,^12^ caused by the highly synchronized firing of neurons with hyperexcitability.^13^ It affects about 70 million population around the world.^14^ Unfortunately, about one‐third of epileptic patients remain drug‐resistant,^15^ leading to a situation that needs a more effective drug target. An increasing number of studies unveiled a significant role of Na+‐K+‐ATPase in epilepsy. Notably, mutation of genes encoding Na+‐K+‐ATPase leads to epilepsy as part of its phenotype. In rodent models of epilepsy, the activity of Na+‐K+‐ATPase was reported to change as well. Pharmacological inhibition of Na+‐K+‐ATPase will cause epileptic seizure in rodents as well. Na+‐K+‐ATPase activating antibody, by contrast, was reported to have a protective effect on epilepsy. However, discordant results are not uncommon, probably due to differences in etiologies, testing timing, features of various epilepsy models, etc. Hence, in this review, we briefly summarize structure and physiological function of Na+‐K+‐ATPase in the CNS. Then we aim to summarize and evaluate current understandings of Na+‐K+‐ATPase and epilepsy, hoping to provide a comprehensive and novel view on the role of ATPase in the epileptic brain and also therapeutic strategies associated with the Na+‐K+‐ATPase. We also provide perspectives on some possible research directions to address the role of Na+‐K+‐ATPase in epilepsy.

## 2 STRUCTURE AND FUNCTION OF Na+‐K+‐ATPase IN THE CNS

Na+‐K+‐ATPase is composed of three subunits, including α, β, and FXYD phenylalanine‐ X‐tyrosine‐aspartate amino acid sequence. One molecule of Na+‐K+‐ATPase contains three molecules of α subunits and two molecules of β subunits.^16^, ^17^ The α subunit consists of four kinds of different subtypes (α1–α4), with the first three being expressed in the CNS. The α1 subtype is expressed in neurons, and glial cells, α2 mainly in astrocytes,^18^ and α3 in neurons.^19^, ^20^ The β subunits are expressed in neurons, astrocytes, and oligodendrocytes. The distribution of each subunit is summarized in Table 1.

**TABLE 1:** Subunits of Na+‐K+‐ATPase in the CNS

Subunit	Function	Subtype	Distribution
α	Catalytic subunit: Ion transportation	α1	Neurons and glial cells
		α2	Astrocytes and oligodendrocytes
		α3	Neurons
β	Assists the newly synthesized α subunit in folding, targeting, and inserting cell membrane correctly	β1	Neurons
		β2	Astrocytes
		β3	Oligodendrocytes
FXYD	Modulates the affinity of α subunit for sodium and potassium	FXYD1	Neurons and astrocytes
		FXYD7	Neurons and astrocytes

The α subunit is the catalytic subunit, containing three envelope domains and ten transmembrane helices. Exporting or importing ions is accomplished by changing two conformational states, E1 and E2. E1 faces the cytoplasm, which has a high affinity for sodium ions. E2 faces extracellular space, with a low affinity for sodium ions but a high affinity for potassium ions.^2^ The function of Na+‐K+‐ATPase is primarily related to the α subunit, which alternates between E1/E2 and phosphorylated E1 (E1‐p)/phosphorylated E2 (E2‐p). By conformational changes of phosphorylation and dephosphorylation, the affinity to sodium and potassium ions also changes.^21^ When the α subunit bounds sodium ions through the E1 state, which has a high affinity to sodium ions, ATP is going to be hydrolyzed. Once one molecule of ATP is hydrolyzed, three sodium ions will be transported to extracellular compartment while two potassium ions to the intracellular compartment,^20^, ^22^ which further raises the resting membrane potential.

The β subunit mainly assists the newly synthesized α subunit with folding, targeting, and correct insertion into the cell membrane,^23^ thereby stabilizing the structure and regulating the activity of the α subunit.^24^ The β subunit includes three isoforms (β1–β3)^25^, ^26^ and is vital in the α subunit to the plasma membrane and functions as an intercellular adhesion protein.^27^ Three of the seven members of the FXYD family were found to modulate the affinity of the α subunit for sodium and potassium ions.^28^ Despite many studies investigating the α subunit, the functions of β and FXYD subunits remain ambiguous.

## 3 Na+‐K+‐ATPase AND SEIZURES/EPILEPSY: EVIDENCE FROM BENCH AND BEDSIDE

### 3.1 Na+‐K+‐ATPase gene mutation leads to epilepsy

Current evidence based on patients with Na+‐K+‐ATPase gene mutation found epilepsy to be a frequent occurrence as phenotypes. In particular, all reported cases with ATPase genetic mutation fall into the sequences encoding the α subunit, with *ATP1A1*, *ATP1A2* and *ATP1A3* corresponding to the four subtypes of α subunit (Table 2).

**TABLE 2:** Mutation of genes encoding Na+‐K+‐ATPase leads to epilepsy

Mutant gene	Mutational point	Epilepsy related syndrome	References
*ATP1A1*	p.Leu302Arg	Repeated SE	^29^
	p.Gly303Arg	Monthly seizures	^29^
	p.Met859Arg	Frequent seizures, repeated SE	^29^
	p.Gly864Arg	Epileptic seizures	^30^
*ATP1A2*	p.Thr378Asn	Febrile and a febrile GTCS	^31^
	p.Gly900Arg	Epileptic seizure	^31^
	p.Met813Lys	Clonic movements or tonic flexion	^40^
	Arg1008Trp	Right‐sided hemiclonic seizures	^82^
	p.Pro364Leu	Febrile seizures	^83^
	p.Asp301Asn	Partial, generalized seizures	^84^
	p.Asn775Ser	Febrile seizures	^85^
	p.His927Arg	Generalized seizures	^85^
	p.Ala378Gly	Generalized seizures	^86^
	p.Tyr1009X	Generalized tonic‐clonic seizures	^33^
*ATP1A3*	p.Glu815Lys	Neonatal‐onset seizures	^87^
	p.Gly358Val and p.Ile363Asn	Catastrophic early life epilepsy	^40^
	p.Phe913del	Focal epileptic seizures	^88^
	p.Cys346Arg	Multifocal epilepsy	^88^

#### 3.1.1 ATP1A1

Schlingmann et al.^29^ reported that heterozygous de novo mutations in *ATP1A1* were found in children diagnosed with hypomagnesemia and epilepsy. These children developed symptoms of convulsions and hypomagnesemia in the infant period from 6 days to 6 months old. All children were treated with antiepileptic drugs and intravenous magnesium. However, seizures remained after a follow‐up of about five years, with some even having status epilepticus (SE), indicating the refractoriness of their epilepsy. Apart from that, some of them also have severe intellectual disabilities. Lin et al.^30^ reported a clinical case of a boy aged 2 years and 10 months with a de novo *ATP1A1* variant. He had severe developmental delay, developed epileptic seizures five months after birth, and later switched into generalized tonic–clonic seizures (GTCS).

#### 3.1.2 ATP1A2

Mutations in the *ATP1A2* gene generally occur in familial hemiplegic migraine (FHM, familial hemiplegic migraine). About 6% of FHM patients have epilepsy and epileptic symptoms typically occur in adolescence.^31^, ^32^ The cases of patient with mutation in *ATP1A2* were reported to be associated with FHM and benign familial infantile convulsions.^33^, ^34^ Moreover, FHM2 is a Mendelian model disease for spreading depolarization, and Clemens Reiffurth et al. showed only α2 heterozygous mice displayed higher spreading depolarization susceptibility,^35^ indicating the α2 subunit is crucial in this disease. In addition, homozygous truncating variants of *ATP1A2* would cause a novel lethal recognizable polymicrogyria syndrome. Polymicrogyria is responsible for a wide range of neurological symptoms, including epilepsy.^36^, ^37^, ^38^ The mechanism under epileptogenicity of polymicrogyria remains unknown. However, unilateral multilobar polymicrogyria is often relevant to an age‐related syndrome of epilepsy.^39^

#### 3.1.3 ATP1A3

Mutations in *ATP1A3* are generally believed to be related to alternating hemiplegia in childhood. Those patients were reported to have concomitant epilepsy (pediatric case of catastrophic early life epilepsy),^40^ especially in those with mutations in p.Glu815Lys, p.Gly358Val, and p.Ile363Asn of *ATP1A3*. In addition, these mutations manifested as a decrease in the activity of the α3 subunit in vitro, which is the basis for these diseases.^41^ Further, Clapcote et al^42^ also presented a mouse model for epilepsy caused by mutation of the Na+‐K+‐ATPase α3‐isoform, which show impairments in the sodium pump and hyperexcitability in the CNS.

Together, in clinical research, mutations in Na+‐K+‐ATPase are closely related to epilepsy. *ATP1A1* codes for the α1 subunit of Na+‐K+‐ATPase, which is responsible for maintaining the membrane potential of neurons.^29^ A primary disease that is relevant to *ATP1A1* mutation is hypomagnesemia and epilepsy. *ATP1A2* gene corresponds with the α2 subunit of Na+‐K+‐ATPase, which is crucial for restoring membrane potential in astrocytes.^43^ *ATP1A2* mutation is generally associated with familial hemiplegic migraine and polymicrogyria in the brain. The α3 subunit of Na+‐K+‐ATPase is encoded by *ATP1A3*, which is vital for maintaining ionic homeostasis in neurons^44^ and responsible for several brain diseases such as alternating hemiplegia in childhood. They all have epileptic symptoms. Previous studies have mainly focused on the clinical spectrum of patients with variants in the ATPase gene. However, effective treatment is still lacking. Targeted interventions using genetic manipulations are promising strategies for treating such diseases, which merit further investigations.

### 3.2 Na+‐K+‐ATPase in an animal model

#### 3.2.1 Change of Na+‐K+‐ATPase activity after epileptiform activity

A large number of studies indicated that Na+‐K+‐ATPase activity decreases in epilepsy.^11^, ^45^, ^46^ In epileptic seizures, abnormal activity of Na+‐K+‐ATPase can be found on the cell membrane of the cerebral cortex of animals and humans.^47^ The decrease of Na+‐K+‐ATPase activity was first reported by Hunt WA et al. in animal models of cobalt‐induced epilepsy, where a significant reduction in enzyme activity was observed.^48^ In the human epileptic brain, the activity of Na+‐K+‐ATPase is significantly less in the epileptic human cortex than that in the nonepileptic cortex.^49^ The decrease of Na+‐K+‐ATPase activity was further corroborated in the model of audiogenic epilepsy,^50^ pentylenetetrazole (PTZ)‐induced seizures in rats,^7^, ^43^ kainate‐lesioned rats^51^ and in the pilocarpine model of temporal lobe epilepsy (especially one hour after SE).^52^ In the freeze lesion cat model, a decreased activity of Na+‐K+‐ATPase was observed three weeks (chronic freeze lesion cat model) after the production of cold lesions.^53^ However, one report found that no significant differences were observed between epileptic mice and normal mice for Na+‐K+‐ATPase activities in various brain regions, including the hippocampus, brain stem, or cerebellum, suggesting seizure susceptibility in epilepsy mice is not associated with differences in the activities of these Na+‐K+‐ATPase activities.^54^

Notably, 60 s after the production of cold lesions in the same model (acute freeze lesion cat model), Na+‐K+‐ATPase activity of the whole brain increases. In addition, in the pilocarpine model of epilepsy, during a chronic period, 120 days after pilocarpine‐induced SE, the activity also increases.^55^ In TLE patient tissue, Na+‐K+‐ATPase in surviving neurons is upregulated.^56^ It is speculated there is a compensatory change resulting from the increased total brain Na^+^.

As mentioned before, the activity of Na+‐K+‐ATPase decreases in most situations. However, in some animal models, the activity increases. Moreover, Reime Kinjo^57^ and Fernandes^52^ reported discordant results, which may be due to the P16 rat used by Reime Kinjo E. From these conflicting data, it could be postulated that under different situations (different stages of epilepsy or different type of epilepsy models), the activity of Na+‐K+‐ATPase would vary. The various changes of Na+‐K+‐ATPase activity in different animal models of seizure/epilepsy are summarized in Table 3 **.**

**TABLE 3:** The activity of Na+‐K+‐ATPase in animal epilepsy model

Model	Na+‐K+‐ATPase functional changes	Specific stage	References
Chronic cobalt‐induced model	↑ ↓(afterward)	5–10 day 10–40 day	^89^
Cobalt‐induced model	↓	2–23 day	^48^
Acute freeze lesions	↑	3–5 h	^53^
Chronic freeze lesions	↓	21 day	
Kainite model	↓	7–21 day	^51^
Pilocarpine induced SE model	↓ (Acute and silent period) ↑(Chronic period)	Acute period:1/24 h Silent period:7 day Chronic period:120 day	^52^
Pilocarpine induced SE model(P16)	↑	7 and 30 day	^57^
Pilocarpine induced SE model	↓	60 day	^90^
Audiogenic seizures in mice	↓	21 day	^50^
Pentylenetetrazol model	↓	15 min	^7^

#### 3.2.2 Epileptic discharge after Na+‐K+‐ATPase inhibition

Inhibition of Na+‐K+‐ATPase will lead to epileptic seizure. Previous studies consistently employed ouabain or [^3^H]Ouabain as an inhibitor of Na+‐K+‐ATPase.^58^, ^59^, ^60^, ^61^ Ex vivo studies showed that 64.5% of ganglion neurons will depolarize after applying ouabain, along with a decrease in input resistance.^62^ Injection of ouabain into the cerebral cortex in vivo can also cause epileptic symptoms, and the symptoms will last for several hours.^61^ It is postulated that the Na^+^‐Ca^2+^ exchange is strengthened after Na+‐K+‐ATPase inhibition. This causes the intracellular Ca^2+^ to increase and leads to an increase in cellular excitability. However, there is currently a lack of specific inhibitors for the different subunit of Na+‐K+‐ATPase, which lead to some unspecific outcome in studies using ouabain.

Using heterozygous knock‐out mice, we could know that deficiency of the α2 isoform of Na+‐K+‐ATPase increases spreading depolarization susceptibility in acute brain slices when exposed to high K+.^35^ Moreover, spreading depolarization has been proposed as a risk factor for the development of epilepsy.^63^

#### 3.2.3 Protective effects in epilepsy after applying Na+‐K+‐ATPase activating antibody

Some researchers use Na+‐K+‐ATPase activating antibody DRRSAb to activate the ion pump. Freitas ML et al. incubated brain slices with DRRSAb and reported a subsequent glutamate release ex vivo.^64^ Furthermore, activation of Na+‐K+‐ATPase using DRRSAb in vivo decreased seizure susceptibility in the pilocarpine model of epilepsy.^64^ Funck et al.^5^ also found that DRRSAb will increase hippocampal Na+‐K+‐ATPase activity in mice and attenuate seizure susceptibility in post‐SE animal models. These results showed that the intervention of Na+‐K+‐ATPase would impact epilepsy.

## 4 Na^+^ ‐K^+^‐ATPase MODULATES CELL EXCITABILITY IN THE EPILEPTIC BRAIN

**FIGURE 1:**
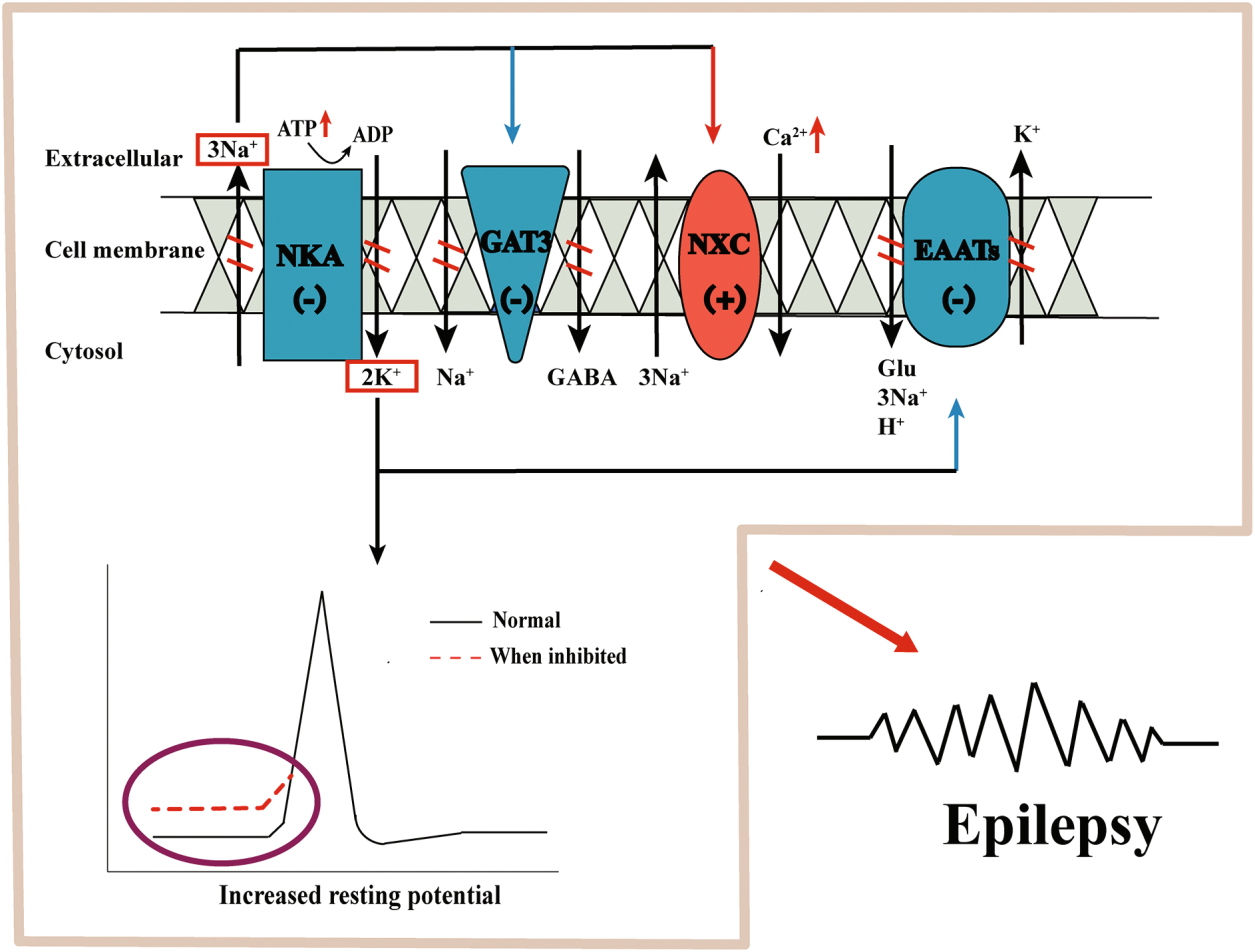
Potential mechanisms of Na+‐K+‐ATPase dysfunction contributed to epilepsy. The inhibition of Na+‐K+‐ATPase could cause the accumulation of intracellular sodium ions, which will further lead to (1) inhibition of inward transportation of GABA; (2) increased NMDA receptor‐mediated excitation; (3) accumulation of intercellular free calcium ions through the reversion of intercellular Na^+^‐Ca^2+^exchanger. Furthermore, Na+‐K+‐ATPase dysfunction is also associated with a decrease in intracellular potassium ion homeostasis, resulting in (1) impaired glutamate clearance; (2) an increase in resting membrane potential. With these mechanisms, Na+‐K+‐ATPase is a major contributor to brain excitability in epileptic brains. NKA, Na+‐K+‐ATPase; GAT3, GABA transporters 3; NXC, Na^+^‐Ca^2+^ exchanger; EAATs, Excitatory amino acid transporters

In the resting state, the cell membrane is much more permeable to potassium than sodium. The equilibrium potential of potassium ions dominates the resting membrane potential. Therefore, Na+‐K+‐ATPase, which determines the potassium gradients across cell membranes, is an essential factor in the maintenance of the resting membrane potential and the regulation of ion homeostasis.^23^ We could understand the mechanisms underlying ATPase and cellular excitability from the perspective of sodium and potassium ions homeostasis, respectively (Figure 1).

### 4.1 Sodium‐ion homeostasis related excitability

#### 4.1.1 Decreased GABA transportation

GABA is the main inhibitory neurotransmitter in the CNS. In particular, GAT3 (GABA transporters 3) is one of the transporters of GABA, which is specifically expressed in astrocytes. The activity of GAT3 depends on the sodium ion gradient.^65^ Therefore, Na+‐K+‐ATPase dysfunction will lead to abnormalities in GABA transportation, thereby increasing CNS excitability.

#### 4.1.2 Dual influence on glycine and its transporter Glyt1b

The dysfunction of Na+‐K+‐ATPase will cause an increase in intra‐astrocytic Na^+^ level. The rise of Na^+^ concentration has a dual influence on the NMDA receptor co‐agonist glycine and its transporter Glyt1b.^66^ It may increase NMDA receptor‐mediated excitation at a low concentration. However, at a higher concentration it may also exert an inhibitory effect on glycine‐mediated activation.

#### 4.1.3 Calcium ion influx

Na+‐K+‐ATPase dysfunction will cause the accumulation of intracellular sodium ions, which will induce the activation of voltage‐gated calcium pumps and the reversion of intercellular Na+‐Ca2+ exchanger, leading to the rapid accumulation of intercellular free calcium ions.^43^, ^67^ The rapid influx of calcium depolarizes the cell membrane due to its divalent positive charge, and it can also mediate action potential firing and potential membrane oscillations.

### 4.2 Potassium ion homeostasis related excitability

#### 4.2.1 Dysfunction of glutamate clearing

Glutamate is the main excitatory neurotransmitter whose transporter in the synapse depends on excitatory amino acid transporters (EAATs). When one molecule of glutamate is transported, one potassium ion is exported, and three sodium ions and one molecule of hydrogen ion are imported. With Na+‐K+‐ATPase dysfunction, glutamate will have clearance impairment, thereby increasing CNS excitability.^68^

#### 4.2.2 Increased resting potential

When the activity of Na+‐K+‐ATPase is inhibited, the active transport between sodium and potassium ions cannot be completed, leading to the accumulation of potassium extracellularly. This will be followed by an increase of resting membrane potential, finally leading to an increase of cellular excitability.

### 4.3 Increased extracellular ATP concentrations

ATP was first proposed as a neurotransmitter by Geoffrey Burnstock et al.^69^ In the CNS, ATP operates as a fast excitatory neurotransmitter.^70^ Some studies reported an increase in extracellular ATP concentration during seizures.^71^, ^72^ With Na+‐K+‐ATPase dysfunction, the concentration of ATP will increase. The elevated concentration of ATP will further activate purinergic P2 receptors,^73^ leading to an exacerbation of seizures.^74^ Among the subtypes of P2 receptors, hippocampal P2X7 receptors were reported to have a selective increase in the intra‐amygdala kainic acid mice,^75^ which further promotes the glutamate release.^76^ However, the specific mechanism underlying P2 receptors and seizures remains unclear.

Although Na+‐K+‐ATPase dysfunction can lead to hyper‐excitability in many ways, the mechanism of Na+‐K+‐ATPase involved in the epileptic brain is still not fully understood. Furthermore, how Na+‐K+‐ATPase in different types of cells, like neurons or glia, contributes to overall excitability is unclear.

### 4.4 Dysfunction of injury‐related autophagy

Brain injury is one of most frequent causes of epilepsy. Brain injury is related to autophagy through Na+‐K+‐ATPase. The interaction between Na+‐K+‐ATPase and autophagy protein Beclin 1 increases when brain have an injury, including ischemia.^77^ Moreover, Na+‐K+‐ATPase dependent autophagy can produce a protective effect on brain injury.^78^ Interestingly, recent studies demonstrated that autophagy also has a close relationship with epilepsy. The abnormal activation of the mTOR pathway, which would impair autophagy, could cause various epilepsy syndromes.^79^ Specifically, conditional deletion of *Atg7*, an essential regulator of autophagy, in mouse forebrain neurons is sufficient to promote development of spontaneous seizures,^80^ the mechanism of which is due to disinhibition of mTOR pathway. In addition, balloon cells in the brains of focal cortical dysplasia (FCD) type IIb patients, a common type of epilepsy, exhibit an increase of autophagy‐related proteins, which indicates autophagy is impaired in FCD. Inhibiting mTOR could reverse this phenomenon.^81^ Therefore, a prospective mechanism may exist in the role of Na+‐K+‐ATPase in epilepsy through the regulation of autophagy, which is worth exploring in the future.

## 5 CONCLUSION AND PROSPECT

Na+‐K+‐ATPase is an ATP‐driven ion transporter pivotal for physiological structure and function of cell activity. It is closely related to cellular excitability, which indicates the vital role of Na+‐K+‐ATPase in epilepsy. The presence of epileptic symptoms in numerous neurological diseases caused by the mutation of Na+‐K+‐ATPase subunits highly suggested the cause‐and‐effect relationship between Na+‐K+‐ATPase and epilepsy. Furthermore, a substantial change in the activity of Na+‐K+‐ATPase was observed in different epilepsy/seizure animal models, although discordant results among studies exits. Pharmacological inhibition of Na+‐K+‐ATPase in rodents will lead to cellular hyperexcitability and seizures. We further highlight the potential mechanisms underlying the role of Na+‐K+‐ATPase dysfunction and cellular hyperexcitability, which is mainly mediated by the change in sodium and potassium ion homeostasis.

However, several questions remain unknown, which mainly revolve around the impact of Na+‐K+‐ATPase on epilepsy and its clinical translational significance.

1. The detailed change of Na+‐K+‐ATPase in epilepsy, including the protein level and subunit alterations, remains elusive. Through investigating the protein level, the direct reason why the activity of Na+‐K+‐ATPase has changed might be explained. Furthermore, the role of each subunit of Na+‐K+‐ATPase in epilepsy must be further clarified. Each subunit of Na+‐K+‐ATPase exists in different cell types. Understanding changes in specific subunits will better address the cell‐specific role of Na^+^‐K^+^‐ATPase in epilepsy. This can assist us in finding new drug targets and explore a series of recent drug structures.
2. In different types and phases of epileptogenesis, the role of Na+‐K+‐ATPase may vary. In clinical, genetic diseases with mutations in the gene encoding Na+‐K+‐ATPase can have different types of epilepsy manifestations. The pathophysiological contribution of Na+‐K+‐ATPase in those patients remains to be further investigated. Moreover, in other types of epilepsy, including TLE with hippocampal sclerosis and FCD, the role of Na+‐K+‐ATPase remains uninvestigated as well. Whether Na+‐K+‐ATPase contributes to epileptic seizure in a common way or via various mechanism in different types of epilepsy needs to be investigated. Further, investigation of the function of Na+‐K+‐ATPase in different phases of epileptogenesis, including acute epileptogenic phase, latency period and chronic spontaneous seizure period, will be of great value in understanding its role in the development of epilepsy.
3. Currently, no one drug that specifically intervene Na+‐K+‐ATPase exists. With specific drugs, the function of Na+‐K+‐ATPase could be evident. Moreover, it would have clinical transformational prospects if such specific drugs could be discovered.
4. The cell‐specific role of Na+‐K+‐ATPase remains largely uninvestigated. The fact that each subunit of Na+‐K+‐ATPase predominates in different cell types might indicate distinct functions of subunits. Previous research failed to identify the cell‐specific role of Na+‐K+‐ATPase in pathological states, including epilepsy. Combined with gene intervention with Cre‐Loxp strategy or subunit‐specific drug with structural virtual screening, the cell‐specific functions of Na+‐K+‐ATPase would have paramount therapeutic implications not only for the treatment of epilepsy, but also other neurological diseases.

## AUTHOR CONTRIBUTIONS

ZC and YW conceptualized the review. JYS and YZ conducted the systematic search and extracted the eligible studies. JYS drafted the review. YW and YZ revised the manuscript. All authors read and approved the final manuscript.

## CONFLICT OF INTEREST

The authors declare no conflicts of interest.

## Data Availability

Not applicable.
